# Nucleic Acid Catalysis under Potential Prebiotic Conditions

**DOI:** 10.1002/asia.201901205

**Published:** 2019-12-09

**Authors:** Kristian Le Vay, Elia Salibi, Emilie Y. Song, Hannes Mutschler

**Affiliations:** ^1^ Biomimetic Systems Max Planck Institute of Biochemistry Am Klopferspitz 18 82152 Martinsried Germany

**Keywords:** catalysis, deoxyribozymes, nucleic acids, origin of life, ribozymes

## Abstract

Catalysis by nucleic acids is indispensable for extant cellular life, and it is widely accepted that nucleic acid enzymes were crucial for the emergence of primitive life 3.5‐4 billion years ago. However, geochemical conditions on early Earth must have differed greatly from the constant internal milieus of today's cells. In order to explore plausible scenarios for early molecular evolution, it is therefore essential to understand how different physicochemical parameters, such as temperature, pH, and ionic composition, influence nucleic acid catalysis and to explore to what extent nucleic acid enzymes can adapt to non‐physiological conditions. In this article, we give an overview of the research on catalysis of nucleic acids, in particular catalytic RNAs (ribozymes) and DNAs (deoxyribozymes), under extreme and/or unusual conditions that may relate to prebiotic environments.

## Graphical abstract

**Many different physicochemical parameters** influence nucleic acid catalysis, including temperature, pH, pressure, dehydration and metal ion composition. In order to explore plausible scenarios for early molecular evolution and investigate to what extent nucleic acid enzymes can adapt to non‐physiological conditions, it is essential to understand how different environmental conditions both inhibit and potentiate nucleic acid catalysis. This review demonstrates that nucleic acid catalysts can tolerate a wide range of challenging environments, and in many cases are enhanced by environmental factors.

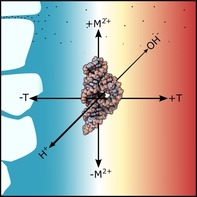

## 1 Introduction

The discovery of the catalytic properties of nucleic acids by Cech and Altman in 1982‐83 both redefined biological catalysis and provided compelling support for origin of life hypotheses centered around nucleic acid‐based information storage and catalysis, in particular the “RNA world” hypothesis first suggested by Alexander Rich, in which self‐replicating RNA emerged prior to the evolution of DNA and proteins.^1^, ^2^, ^3^ Despite the prevalence of the RNA World hypothesis and related conjectures, such as different “pre‐RNA” worlds^4^ and mixed chimeric systems including, for example, both RNA and DNA,^5^ a key unanswered question is: under which environmental conditions did functional nucleic acids emerge and sustain themselves? Constraining the parameter space of a habitable early Earth is crucial to understanding the emergence of life. One way of achieving this is to consider the sensitivity of nucleic acids to environmental conditions: in what conditions can nucleic acids survive, and do conditions exist which can potentiate nucleic acid catalysis? Exploring conditions more exotic than dilute buffered solutions may yield answers to intractable problems in origin of life and synthetic biology research.^6^, ^7^

**Figure 1:**
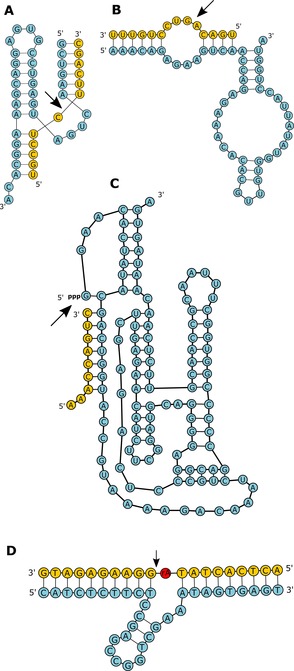
Secondary structures of various nucleic acid enzymes, including the hammerhead ribozyme, hairpin ribozyme, the class I ligase and 8–17 DNAzyme. The hammerhead (A) and hairpin (B) ribozymes catalyze the reversible cleavage of the RNA substrate strand shown in yellow (black arrow indicates cleavage site).^42^ The class I ligase (C) binds a substrate strand (yellow) and catalyzes 3′ OH nucleophilic attack on its own 5′ triphosphate, leading to phosphodiester bond formation and release of inorganic pyrophosphate.^43^ The 8–17 DNAzyme (D) is a metalloenzyme catalyzing RNA transesterification in the presence of divalent metal ions.^44^ The substrate strand is shown in yellow, with the ribonucleotide cleavage site marked in red.

A wide range of catalytic nucleic acids are known today. For RNA (ribozymes), the most iconic example is the ribosome,^8^ whose central role in peptide bond formation and thus protein synthesis designates it the most important ribozyme in modern biochemistry, and the most obvious “smoking gun” of an early RNA world predating modern biochemistry. Another ubiquitous ribozyme that is essential in all free‐living organisms is RNAseP, which processes the 5′‐ends of precursor‐tRNAs.^9^, ^10^ Other prominent examples for ribozymes are small RNA‐cleaving ribozymes such as the hammerhead (HH) ribozyme^11^, ^12^ (Figure 1 A) and the hairpin (HP) ribozyme^13^ (Figure 1 B), which catalyze reversible self‐cleavage to process the concatemeric products of rolling circle RNA replication into linear and circular RNA molecules.^14^ A related function is carried out by self‐splicing introns,^15^, ^16^ which catalyze their own excision from messenger, transfer, or ribosomal RNA via two sequential transesterification reactions of the phosphodiester backbone. In addition, in vitro selection experiments have revealed that the palette of RNA catalysis is far broader than these reactions and encompasses RNA ligation,^17^, ^18^ aminoacyl transfer, porphyrin metalation^19^ and C−C bond formation including the Diels–Alder reaction,^20^ Michael addition,^21^ aldol condensations^22^ and others,^23^ suggesting that an early metabolism might have been sustained by ribozymes.

While the main function of DNA in biology is the storage of genetic information, a large number of artificial DNA catalysts have also been isolated by in vitro selection. These deoxyribozymes, or DNAzymes, catalyze a range of bond forming reactions, including the Diels–Alder reaction,^24^ Friedel–Crafts reactions,^25^ RNA ligation (2′‐5′ and 3′‐5′),^26^, ^27^ DNA ligation,^28^ 5′‐phosphorylation,^29^ adenylation,^30^ RNA‐nucleopeptide linkage^31^ and porphyrin metalation.^32^ The full range of DNA catalysis is reviewed in detail by Hollenstein, and an example of a RNA cleaving DNAzyme is shown in Figure 1 D.^33^

Finally, synthetic nucleic acids are also capable of catalysis. In particular, Taylor et al. selected artificial endonuclease and ligase enzymes from random pools of arabino nucleic acid (ANA), 2′‐fluoroarabino nucleic acid (FANA), hexitol nucleic acid (HNA) and cyclohexene nucleic acid (CeNA).^34^

While these studies convincingly demonstrate the broad catalytic potential of polynucleotides, they leave open the question of whether some of these reactions could have contributed to early biocatalysis, and whether they are compatible with the environmental conditions on early Earth.

Since the beginning of the Origin of Life field, great efforts have been made to determine, or at least constrain, the conditions under which life originated. Definitive answers have been elusive, due to the extreme timescales under consideration and the combined uncertainties of when, where and how the first primitive forms of life emerged. The lack of fossil evidence of early life, the large number of possible geochemical environments and the difficulty in determining conditions on early Earth make this an almost intractable problem for origin of life researchers, amongst whom there is little consensus on these questions.^35^, ^36^ In light of this, we and others have previously argued for a flexible approach to the problem, by performing experiments under relaxed but plausible boundary conditions and using the results to inform about possible plausible prebiotic environments.^37^, ^38^, ^39^, ^40^, ^41^

The many studies that aim to constrain the global climate and conditions on early Earth allow some experimental boundaries to be set: As today, divalent magnesium and calcium were abundant in the oceans of early Earth. Historical ocean solute composition is dependent on both pH and reducing potential. Assuming an acidic ocean pH around 4 Ga, hydrogen sulfide present in seawater would have created a reducing environment rich in Fe^2+^, but low in concentrations of free transition metal and group 12–16 ions due to the formation of insoluble sulfide compounds.^45^, ^46^ Early nucleic acid catalysis may have relied on Fe^2+^ as a cofactor, until the advent of aerobic conditions caused the oxidation of Fe^2+^ to Fe^3+^, necessitating its replacement by Mg^2+^ or other metal ions.^47^ Oceanic pH, which is driven by atmospheric CO_2_ concentrations, likely rose monotonically from pH 6.6 in the Hadean era to pH 7.9 by the Cambrian era.^48^ However, other studies posit that oceanic pH in the late Hadean/ early Archean was as low as 3.5–5.4.^49^, ^50^ Further uncertainty is introduced if we consider that life may have emerged in the vicinity of a hydrothermal vents, where local pH may be either very low (pH 2–3) or very high (pH 9–11), depending on type, rather than in the bulk ocean.^51^

Estimates of temperature are more variable, spanning climates ranging from frozen to near boiling. Oxygen, iron and silicon isotope studies suggest temperatures of 70 °C up until as late as 3.3 Ga, a theory additionally supported by evidence of a low viscosity Archean ocean.^52^, ^53^, ^54^, ^55^ However, evidence of a temperate climate is provided by geological carbon cycle models and isotope evidence from cherts and sediments.^56^, ^57^, ^58^ Studies of Archean glacial deposits suggest the presence of ice caps or cold periods during this time,^58^ and some researchers argue that in the absence of extreme levels of greenhouse gases, a glacial Hadean Earth is likely, albeit with intermittent periods of “fire and brimstone” following major impacts.^59^, ^60^

Although these studies provide some useful constraints on the conditions at the Origin of Life, a broad range of conditions remain feasible. The exact microenvironment in which the first replicators emerged was likely more significant than the global conditions at the time. For example, ‘warm little ponds’ on land would be subject to temperature, composition and concentration fluctuations due to evaporation and condensation driven by day–night cycles,^61^ eutectic phases in frozen environments lead to strong solute up‐concentration and significant pH shifts,^62^ and hydrothermal vents provide extreme temperature and pH gradients.^51^ Any of these environments might provide shelter from adverse conditions such as UV radiation, the surface intensity of which was several orders of magnitude higher than today.^63^

In this focus review, we will explore the range of conditions under which nucleic acid catalysis is possible, highlighting how nucleic acids can adapt to extreme conditions, and how these conditions can both support and potentiate function. In order to understand the emergence of life, we must understand the environmental factors that would have acted upon the first functional nucleic acids, for example, in an RNA, proto‐RNA or mixed nucleic acid world scenario. In addition, many nucleic acid enzymes catalyze industrially relevant processes and, as such, challenging conditions may be required to increase reaction rates, shift reaction equilibria or improve substrate or product solubility. In both cases, reaction conditions may deviate strongly from in vivo or typical in vitro environments.

## 2 The role of metal ions in nucleic acid folding and catalysis

### 2.1 Folding of nucleic acids

The range of conditions in which catalytic nucleic acids are functional is largely determined by the mechanism by which nucleic acids can fold into catalytically active three‐dimensional structures. Nucleic acid folding differs to that of proteins, which in many cases tend to fold via rapid, cooperative two‐state thermodynamic transitions, with no detectable intermediate structures.^64^ Nucleic acid chain compaction is driven by ion‐mediated electrostatic interaction, conformational entropy, base pairing, base stacking, and noncanonical interactions.^65^, ^66^ Compared to proteins, the folding energy landscape of nucleic acids is convoluted due to the high number of competing, energetically similar folding states, and nucleic acid molecules tend to adopt a range of conformations in solution.^67^, ^68^ The highly charged polyanionic backbone of nucleic acids usually prevents the irreversible aggregation of misfolded molecules. This means that, whilst activity may be lowered by adverse environmental conditions due to the presence of inactive or poorly active conformers, catalysis can occur under a broad range of environmental conditions. Consequently, conditions that promote folding and the formation of active conformations are of particular interest, as they may directly improve the catalytic activity of nucleic acid enzymes.

### 2.2 Modes of metal ion—nucleic acid interaction

**Figure 2:**
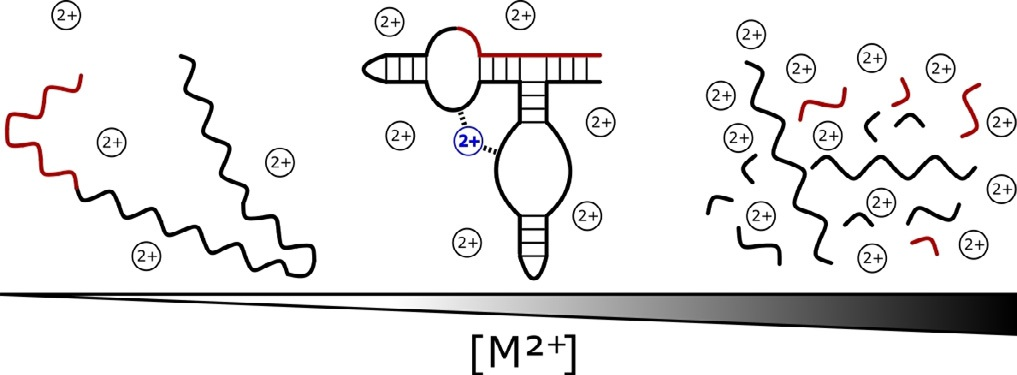
Schematic depicting dependence of RNA folding and hydrolysis on divalent metal ion concentration. Under aqueous conditions, divalent metal ions (in particular Mg^2+^ and Mn^2+^) can enhance RNA folding by both diffuse binding and site‐specific binding (highlighted in blue). In diffuse binding, hydrated Mg^2+^ ions interact nonspecifically with the nucleic acid via long‐range electrostatic interactions. In site binding, dehydrated or partially dehydrated Mg^2+^ ions (highlighted in blue) interact specifically with anionic binding sites, which are formed by the RNA fold to act as coordinating ligands for the metal ion. At high M^2+^ concentrations, metal ion catalysis leads to increased RNA hydrolysis.

A key variable determining nucleic acid folding and activity is the presence of counterions, which help to overcome the charge repulsion from the polyphosphate backbone during compaction. For RNA, the most relevant cations under in vivo conditions are Mg^2+^ and K^+^, both of which interact with RNA predominantly through electrostatic forces.^69^ In particular, Mg^2+^ ions enable the formation of complex folds that allow nucleic acids to stabilize specific structures, recognize binding partners and mediate catalytic processes.^70^, ^71^, ^72^, ^73^ Generally, interacting Mg^2+^ can be divided into two populations (Figure 2): diffusive ions, which surround the RNA as an ensemble of hydrated ions that are non‐specifically attracted to the negative charge of the RNA, and a much smaller group of partially desolvated ions, which bind to specific electronegative sites on the RNA itself.^74^ Whilst these specific metal ion‐RNA interactions mostly contribute to the conformational specificity of an RNA structure (and thus in many cases to the active conformation of nucleic acid enzymes), diffusive ion‐RNA interactions contribute most to the thermodynamic stabilization of the overall RNA fold.^75^

### 2.3 Impact of metal ions on nucleic acid catalysis

Given that magnesium is the seventh most abundant element in the Earth's crust, and that the Mg^2+^ ion is the second most abundant cation (55 mm) in sea water after Na^+^, it is conceivable that similar Mg^2+^ concentrations were present in an Archean ocean,^76^ or at varying levels in potential RNA world freshwater environments. However, many other mono‐, di‐ and polyvalent ions can also drive the folding of RNA (and other nucleic acids), including Mn^2+^, Ca^2+^, Fe^2+^, Sr^2+^, Ba^2+^, Na^+^ and polyamines.^66^, ^77^, ^78^ The ion concentrations required to achieve RNA folding vary between the different ion types, as their charge density and excluded volume largely determine the strength of the coulombic RNA‐ion interaction and thus the overall compactness of the folded nucleic acid.^78^ For example, the *Tetrahymena* group I ribozyme, which was derived from a self‐splicing *Tetrahymena* preribosomal RNA and catalyzes a reaction mimicking the first step of splicing,^79^ requires micromolar concentrations of trivalent cations, millimolar concentrations of divalent ions but near‐molar concentrations of monovalent ions for folding.^75^ However, although the *Tetrahymena* group I ribozyme folds into a native‐like state in the presence of various counterions, folding of the catalytically active state requires site‐specific binding of Mg^2+^ or Mn^2+^.^75^

All of the larger natural RNA enzymes, such as RNAseP^9^, ^10^ and the various self‐splicing introns,^15^, ^16^ depend on site‐specific metal ion cofactors for chemical reactivity. Likewise, the various artificial RNA ligase and polymerase ribozymes, which rely on nucleoside triphosphate activation chemistry, are strict metalloenzymes with only poor tolerance towards metal ions other than Mg^2+^.^80^ In view of this, it is quite surprising that modern intracellular conditions are somewhat challenging for nucleic acid folding and activity due to low free Mg^2+^ concentrations of approximately 1 mm.^81^ The need for higher levels of free Mg^2+^ in vivo is alleviated by the presence of RNA chaperone proteins, which promote RNA folding and annealing.^69^ The dependence on intracellular protein co‐factors is well illustrated by RNAse P: at low ionic strength, the protein component of this complex is essential for activity in vivo and in vitro.^82^, ^83^ However, the RNA itself is active in vitro in the presence of 60 mm MgCl_2_.^2^ The high divalent ion concentration required for RNA‐only catalysis in vitro emphasizes that charge screening by either salt or the protein component is essential for folding and activity. Nevertheless, optimal conditions are highly dependent on the catalytic system in question. For example, the family of group II introns has a broad tolerance for Mg^2+^ concentrations and near‐optimal activity occurs between 0.1 to 100 mm in vitro.^84^

Like ribozymes, DNAzymes use diffuse electrostatic and specific metal ion interactions for activity and folding. Notably, the high stability, cost‐effective production, and easy chemical modification of DNA has enabled the systematic selection of a large number of DNAzymes and aptamers capable of selective metal ion detection. These DNAs can bind to and distinguish between an impressive range of species, including alkali metal ions, alkaline earth metal ions, transition metals, noble metals, post‐transition metal ions and lanthanide and actinide ions for catalysis.^85^

It should be mentioned that non‐metallic ions can also support folding of nucleic acids into active conformations. For example, polyamines can aid RNA folding; the required MgCl_2_ concentration for RNAseP RNA folding and activity is reduced from 60 mm to 10 mm in the presence of 1 mm spermidine.^2^ However, enhancements in folding are dependent on the characteristics of the polyamine counterion. Longer polyamines destabilize folded structures due to excluded volume effects, which can prevent a complete folding transition to the native state even under usually favorable folding conditions.^77^

**Figure 3:**
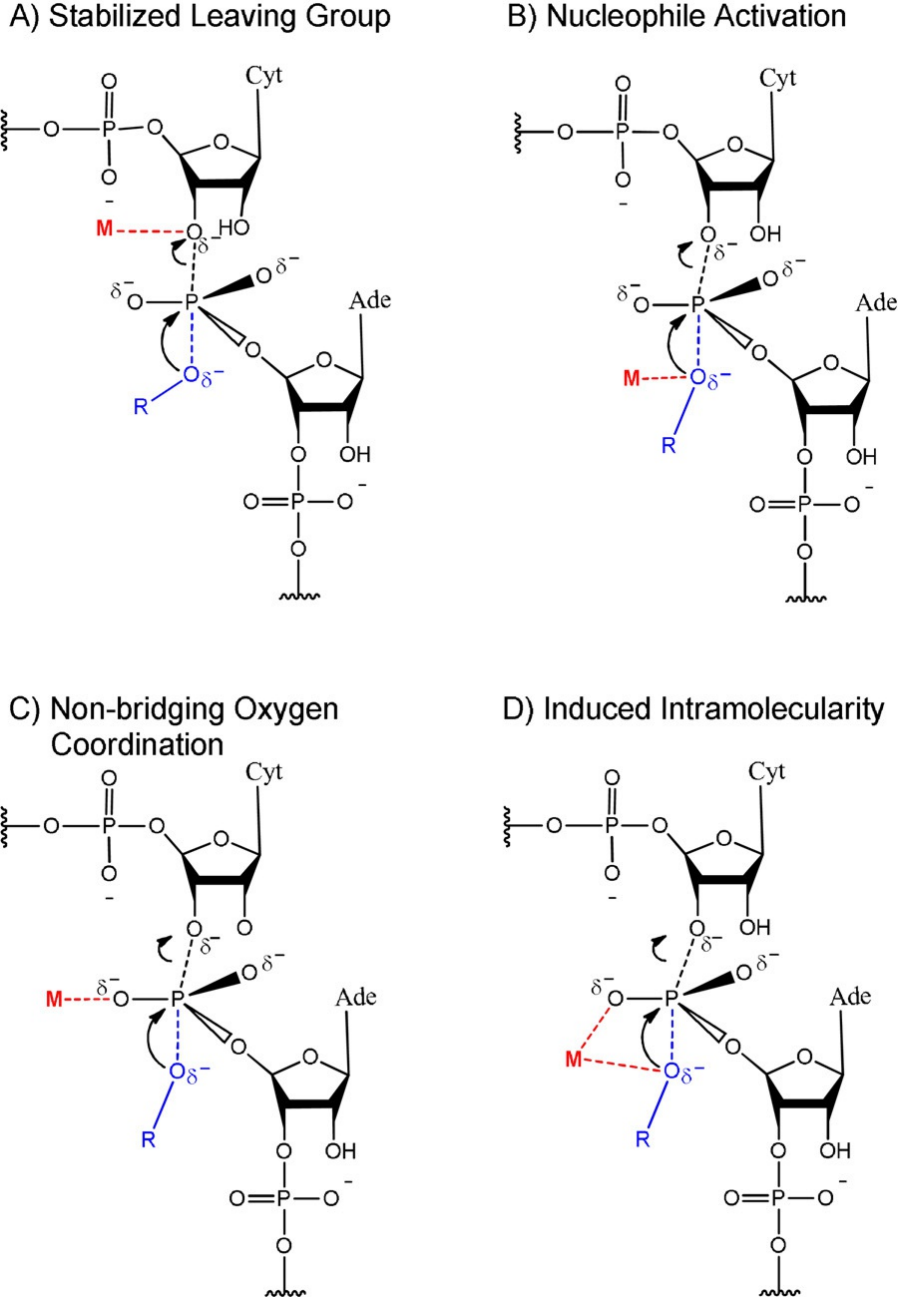
Various modes of interaction between metal ions and RNA during RNA cleavage. The reaction proceeds via a trigonal bipyramidal transition state. The rate of reaction can be accelerated by Lewis acid stabilization of the leaving 3′ oxygen (A), facilitating the deprotonation of the attacking oxygen nucleophile (B), coordination of non‐bridging oxygens (C) or coordination of a non‐bridging oxygen in addition to the nucleophile (D), which promotes a favorable in‐line geometry for nucleophilic attack. The stabilizing metal ion and attacking base are shown in red and blue, respectively. Adapted from Forconi et al. and Frederiksen et al.^104^, ^108^

Lanthanides (Ln^3+^) are also of interest, as their interactions with nucleic acids are very different from typical divalent metal ions due to their unusual coordination chemistry. In particular, the absence of a strong ligand field allows for a high degree of structural diversity in lanthanide complexes, as ligands alone dictate the symmetry and coordination of complexes.^86^ As a result, lanthanides not only show a high affinity to the phosphate backbone of nucleic acids due to their high charge density (typically only μm concentrations are required for binding), but they can also directly interact with the nucleobase moieties.^87^ Because of these unusual properties, the impact of lanthanides on nucleic acid catalysis is rather diverse: Ln^3+^ ions can accelerate a small Pb^2+^‐dependent ribozyme called the leadzyme,^88^ yet they inhibit the hammerhead^89^ and hairpin^90^ ribozymes, and the RNA‐cleaving 8–17 DNAzyme.^91^ In addition, several strictly Ln^3+^‐dependent RNA‐cleaving DNAzymes were discovered by in vitro selection experiments,^92^, ^93^, ^94^, ^95^ suggesting that nucleic acid enzymes can directly harness the Lewis acid character of lanthanides for catalysis (Figure 3). To the best of our knowledge, Ln^3+^‐specific ribozymes have not yet been described in literature, and at a first glance rare earth metals have little relevance for origin of life scenarios due to their low aqueous solubility. However, low concentrations of lanthanides are available, for example, under hot acidic conditions in volcanic mudpots, and Ln^3+^ ions are essential under these conditions for some acidophilic microbes that use methane as an energy source.^96^ This raises the possibility that prebiotic systems relying on nucleic acid catalysis may have been able to harness lanthanides for certain reactions.

### 2.4 Metal ion induced hydrolysis

While metal ions assist nucleic acid folding and catalysis in many cases, they are often also a threat to the chemical integrity of RNA (Figure 2);^97^ heavy metal ions such as Eu^3+^, La^3+^ and Tb^3+^, Pb^2+^, and Zn^2+^ catalyze rapid RNA cleavage in aqueous solutions.^97^, ^98^ Zn^2+^ is only about 4 % as active as Pb^2+^, and other metal ions such as Cd^2+^, Mn^2+^, Cu^2+^ or Mg^2+^ catalyze degradation one to two orders of magnitude slower than Zn^2+^.^99^ However, at elevated temperatures and/or high ion concentrations, these seemingly weak catalysts (including Mg^2+^) can reduce RNA half‐lives down to minutes.^100^ This means that environments with a high concentration of Mg^2+^ and high temperatures, such as hydrothermal vents, are unsuitable settings for RNA‐based scenarios of molecular evolution. Likewise, free Ln^3+^ ions are highly nucleolytic under basic conditions, as their ions form multinuclear complexes and cleave RNA nonspecifically at low mm concentrations with a rate acceleration as large as 10^8^–10^12^‐fold.^101^ DNA is much more resistant towards metal ion‐induced scission, and requires additional DNA‐binding delivery agents for efficient cleavage under mild aqueous conditions.^102^ A notable exception is the ability of Ce^IV^ to accelerate DNA hydrolysis up to 10^11^‐fold under neutral conditions, reducing the half‐life of the phosphodiester linkage in DNA from millions of years down to a few hours.^101^

Possible modes of metal ion‐catalyzed nucleic acid hydrolysis include Lewis acid catalysis, Brønsted base catalysis, nucleophilic catalysis by metal‐bound hydroxides and simple electrostatic stabilization of transition states by positively charged metal ions (Figure 3). The individual mechanisms of each metal ion class are still the subject of some debate and go beyond the focus of this review, but are discussed in excellent detail elsewhere.^101^, ^103^, ^104^

Facing the threat of degradation by metal ions, in particular in the case of RNA, it is interesting from a prebiotic perspective that a number of nucleic acids are capable of efficient catalysis without divalent metal ions. In particular, several families of small nucleolytic ribozymes reversibly catalyze metal‐independent and site‐specific cleavage/ ligation of the RNA backbone, and can accelerate this reaction by approximately a million‐fold using general acid base catalysis.^105^ Similarly, purely Na^+^‐dependent DNAzymes were isolated by targeted in vitro selection.^106^, ^107^ Some of these (deoxy‐)ribozymes will be discussed later in more detail, as they are compatible with a wide range of conditions.

### 2.5 Prebiotic alternatives to Mg^2+^

Of the various ions that can replace Mg^2+^ during nucleic acid folding and catalysis, Fe^2+^ is of great prebiotic interest as it was likely to be highly abundant on Earth before the advent of photosynthesis.^31^ Fe^2+^ was speculated to be present in micro‐ to low millimolar quantities during early Archean Earth.^31^ Such concentrations are sufficient to replace Mg^2+^ during RNA cleavage catalyzed by several DNAzymes.^109^ As discussed in section 3, Fe^2+^ was used during pH‐dependent selection for RNA‐cleaving ribozymes, where it enabled the discovery of novel catalytic motifs that are absent in typical selections using Mg^2+^.^110^ Intriguingly, Hsiao et al. showed that substituting Mg^2+^ with Fe^2+^ in an anoxic environment enabled various natural RNAs, such as tRNA or ribosomal RNA, to catalyze single‐electron transfer reactions, which are typically limited to cofactor‐dependent protein enzymes.^111^ Thus, RNA might have catalyzed different electron transfer reactions, which are a prerequisite for metabolic activity, before the rise of oxygen levels.

Zn^2+^ has also been proposed as a key divalent transition metal ion in prebiotic chemistry.^112^ In this “Zinc World” hypothesis, porous and photoactive structures comprised of ZnS provided the substrate upon which CO_2_ reduction and biomolecular polymerization occurred, driven by UV light. Indeed, Zn^2+^ can substitute Mg^2+^ as the only divalent metal ion during RNAseP catalysis, but only in the presence of high concentrations of ammonium salts.^113^ Zn^2+^ was also shown to be strongly beneficial for DNA‐catalyzed DNA cleavage. The artificial deoxyribozyme 10MD5 is a bimetallic metalloenzyme (analogous to many protein DNA endonucleases) that catalyzes the Mn^2+^/Zn^2+^‐dependent DNA phosphodiester bond hydrolysis with at least a 10^12^‐fold rate enhancement.^114^ In a follow‐up study, Silverman and co‐workers demonstrated that only two base substitutions were necessary to alter 10MD5 from heterobimetallic to a purely Zn^2+^‐dependent monometallic DNAzyme.^115^ Later, even faster and smaller deoxyribozymes which require Zn^2+^ alone for catalysis were identified by in vitro selection.^116^

In summary, the availability of metal ions such as magnesium was most likely not a critical factor for early nucleic acid enzymes (especially ribozymes). However, it is possible that Fe^2+^ ions in particular extended the catalytic properties of ribozymes under the anoxic conditions of the late Hadean and early Archean. Further research in this field could uncover new, unexpected catalytic nucleic acids that increase the plausibility of an early metabolism mediated by nucleic acids.

## 3 The influence of pH on folding and catalysis

### 3.1 Potential pH values in prebiotic settings

**Figure 4:**
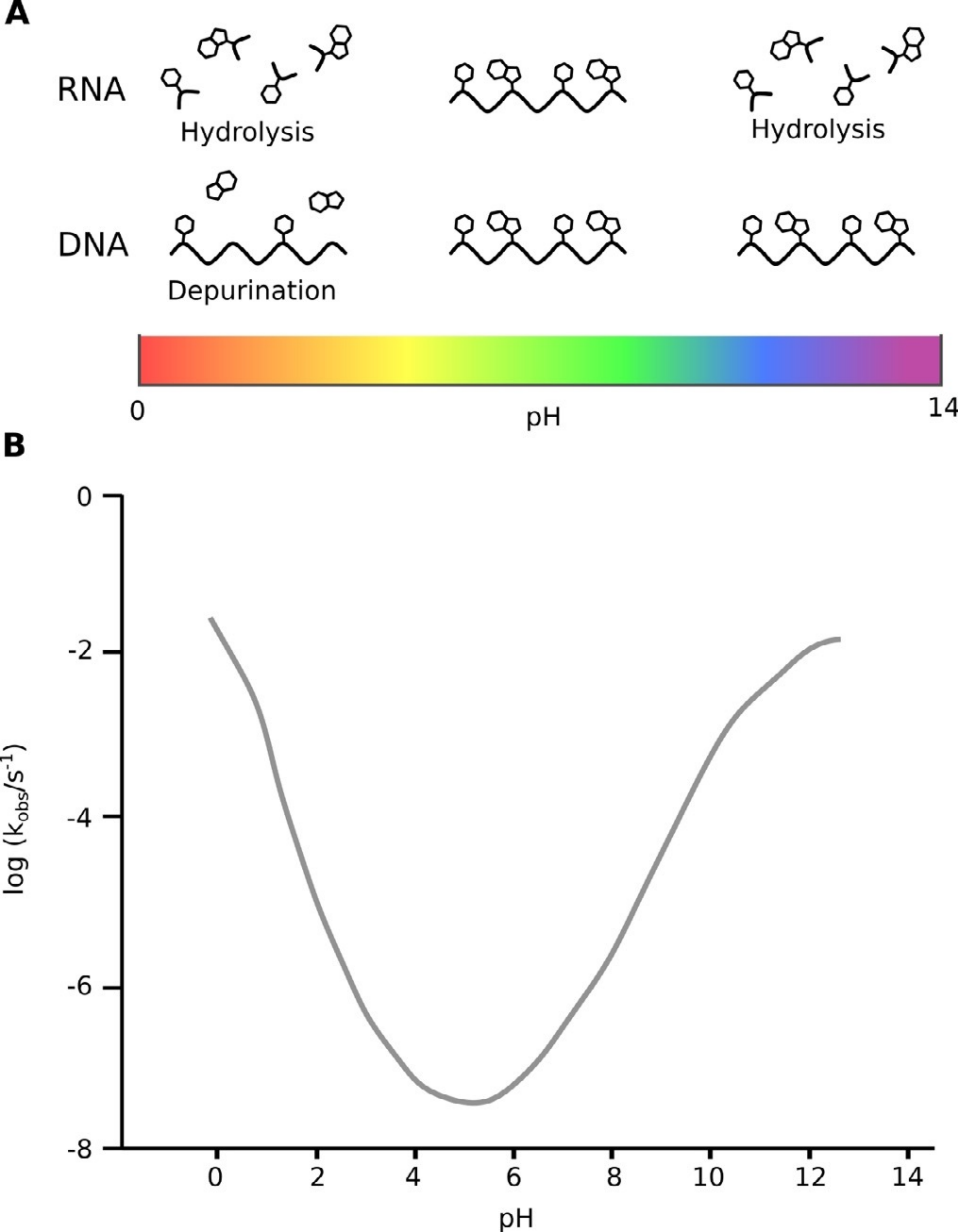
The impact of pH on RNA/ DNA stability. A) Illustration of RNA and DNA stability in different pH ranges. At acidic pH<2, RNA is prone to hydrolysis, whereas DNA is more susceptible to depurination. At basic pH, the phosphodiester backbone of RNA hydrolyses rapidly, whereas DNA remains stable. B) Relative rate of RNA hydrolysis with respect to pH. Shown is an illustrative pH‐rate profile for the cleavage of 3′,5′‐UpU at 90 °C based on the data reported by Jarvinen et al.^131^

Another crucial physicochemical parameter for early nucleic acid catalysis and stability is pH. Estimates of environmental pH on early Earth are largely hypothetical (see introduction), but most evidence suggests that oceanic pH was initially acidic (pH 6.6,^48^ or lower^49^, ^50^).The theory that early molecular evolution originated at alkaline (pH 9–11) hydrothermal vents, similar to the modern Lost City systems, has a number of proponents, but is difficult to reconcile with an RNA‐based origin due to the inherent lability of RNA to alkaline hydrolysis, which occurs above pH 6 and is strongly accelerated by higher temperatures and divalent metal ions (Figure 4).^100^, ^117^ RNA is most stable at pH 4–5 with significant acid hydrolysis not occurring until below pH 2. Thus, more acidic vent types such as acidic volcanic lakes or comet ponds are credible early scenarios for RNA formation and catalysis.^51^

DNA is less stable than RNA under acidic conditions due to increased depurination below pH 3,^118^, ^119^ but is more resistant to basic conditions as it does not possess the 2′‐OH group required for base‐catalyzed hydrolysis. A DNA‐later scenario could therefore be in agreement with a gradual increase of environmental pH over time. Indeed, high CO_2_ levels in the Hadean era may have led to a variety of acidic aqueous environments,^49^ and the slow transition from acidic to slightly alkaline oceans could have driven the later emergence of the more stable DNA‐based systems.^48^, ^120^, ^121^

### 3.2 The impact of pH on nucleic acid catalysis.

**Figure 5:**
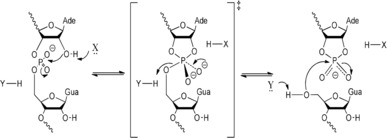
Mechanism of general acid/base‐catalyzed RNA phosphodiester cleavage and ligation. General acid‐base RNA cleavage and ligation catalyzed by nucleolytic ribozymes. In the cleavage reaction (here, a scissile bond between A and G), the 2′‐O attacks the 3′‐P in an SN2 process (left). This leads to the formation of a trigonal bipyramidal phosphorane that is probably close to the transition state (middle). Concurrent breaking of the bond to the 5′‐O leads to a cyclic 2′,3′ phosphate and 5′‐O products. In the ligation reaction, the 5′‐O nucleophile attacks the P of the cyclic phosphate. A general base (X) assisting in the removal of the proton from the 2′‐OH, and a general acid (Y) protonating the 5′‐*O*‐oxyanion leaving group catalyze the cleavage reaction. In the reverse ligation reaction, X and Y act as general acid and base, respectively.

The direct effect of pH on catalysis is inherently dependent on the type and mechanism of the reaction. Catalysis by nucleic acids can occur via transition state stabilization (e.g. by hydrogen bonding or electrostatic stabilization), general acid and/or base catalysis (i.e. by enhancing the nucleophilicity of attacking groups by deprotonation or by stabilizing leaving groups by protonation), or by facilitating active conformational states such as the formation of an in‐line transition state during nucleophilic attack.^122^ For example, the reversible RNA cleavage reaction catalyzed by small nucleolytic ribozymes, which is based on the nucleophilic attack of an O2′ on an adjacent phosphorus atom, is in most ribozymes accelerated by general acid‐base catalysis.^122^ Here, two ionizable groups stabilize the developing negative and positive charges during the reaction by partial proton transfer in the trigonal bipyramidal phosphorane transition state of the reaction (Figure 5).^122^, ^123^ Typically, optimal proton transfer in enzymes requires functional groups with a p*K_a_* in the neutral range.^124^ However, the free form of the four canonical nucleobases have p*K_a_* values far from neutrality and are therefore suboptimal for general acid‐base catalysis.^125^ In some ribozymes, the local molecular environment can cause a considerable shift in the p*K_a_* of both general acid and base towards neutrality, a similar effect to that found in some proteins.^126^, ^127^, ^128^ If both ionizable groups are sufficiently perturbed, the pH dependence of catalytic rates shows a “bell‐shaped” pH rate profile, where the rates are maximal around pH 7.^123^, ^129^ In other cases, such as for the hairpin (HP) ribozyme, the rates of RNA cleavage (and ligation) increase up to pH 7, but plateau at higher values due to the high p*K_a_* of N1 in the catalytically active guanosine base.^130^

Generally, the acid‐base mechanism employed by small ribozymes makes them robust towards changes in pH and enables significant cleavage and ligation activity at pH >6. However, the rate enhancement is limited by the small fraction of ribozymes that, on average, have the correct ionization state for general acid‐base catalyzed cleavage (typically 1 in 10^5^ to 10^6^ ribozymes for the HP ribozyme at neutral pH^122^). For the reverse ligation reaction the inverse ionization state is more favored, but the resulting rates are offset by a low *k*_cat_ due to the low reactivity of the neutral base moieties.^123^

The phosphotransfer reactions of large metalloribozymes such as self‐splicing introns,^132^, ^133^ RNAseP and artificial ligases that make use of triphosphate activation chemistry, show a log‐linear relationship between the rate of the chemical step and pH.^134^ This is typical for a reaction mechanism involving a pre‐equilibrium loss of a proton from a hydroxyl group before in‐line nucleophilic attack. Likewise, most RNA‐cleaving deoxyribozymes have a log‐linear dependence of rate on pH with a slope near unity,^44^, ^135^ which is also consistent with the requirement for a single deprotonation event during the reaction.

pH levels also have an important effect on nucleic acid base pairing, as the protonation state of nucleobases dictates their ability to form hydrogen bonds. In particular, at low pH most nucleic acids are denatured (or at least destabilized) due to the protonation of G‐C base pairs and resultant Hoogsteen base pair formation.^51^ While this mechanism is detrimental for nucleic acid folding, for example, of active ribozymes, environmental pH cycles or gradients^136^ may have lowered DNA and RNA duplex melting temperatures, and therefore facilitated non‐enzymatic and enzymatic copying reactions.^137^ Furthermore, non‐canonical A−C and C−C base pairs have been shown to occur under mildly acidic conditions, with A−C base pairs at pH 5 reaching the stability of A−U and G−U base pairs under neutral conditions.^138^ Thus, different pH regimes can enable the exploration of structural motifs and thus catalytic sequences that are otherwise inaccessible at neutral pH.

### 3.3 In vitro selection of nucleic acids catalysts under non‐physiological pH conditions

Indeed, in vitro selection experiments have shown that nucleic acids can be readily evolved towards improved catalysis at lower pH where the chemical stability of the RNA backbone is strongly increased. For example, a de novo selection of self‐cleaving ribozymes at low pH resulted in a variant that showed pH‐dependent kinetics with an optimum of around pH 4.^139^ Another study by Popović et al. investigated the effects of both pH and divalent cations on the isolation of self‐cleaving RNA in iterative in vitro selection experiments from random libraries.^110^ Depending on pH, and whether Mg^2+^ or Fe^2+^ was included as the divalent metal ion during selection, different sequences and secondary structure motifs were isolated. Neutral pH in the presence of Fe^2+^ led to the selection of hammerhead (HH)‐like motifs, whilst at pH 5 a variety of previously unknown motifs were discovered and the abundance of HH motifs dropped to less than 0.1 %. Thus, both pH and substitutions between Fe^2+^ and Mg^2+^ strongly influence the relative fitness of different motifs.

Short RNA‐cleaving DNAzymes have also been evolved to function in trans at low pH. The reaction proceeds optimally at pH of 4–4.5 in the absence of Mg^2+^, demonstrating that low pH can facilitate the Mg^2+^‐free cleavage of RNA by a DNAzyme.^140^ Moreover, of the 20 clones sampled after selection, 14 did not share extensive sequence similarities, suggesting that the catalysis of the cleavage reaction at low pH has different or relaxed sequence requirements.

Ligation reactions represent an important catalytic function, for example, for nucleic acid self‐replication.^141^ Consequently, RNA ligases have also been evolved to function at acidic pH. For example, random mutagenesis of a derivative of the triphosphate‐dependent class I RNA ligase ribozyme (Figure 1 C), followed by four rounds of evolution of the randomized pool under acidic pH, allowed for the selection of clones that function optimally at pH 4 instead of at neutral conditions.^142^ Additional mutagenesis of the selected ribozyme further enhanced the rate of ligation by 8000‐fold.^143^ Kühne and Joyce implemented a continuous in vitro evolution strategy to progressively decrease or increase the optimal pH of the class I ligase ribozyme, beginning with an optimal pH of 8.5.^144^ The result was two highly active class I ribozyme variants with only very few mutations that shifted the optimal pH to either pH 5.8 or 9.8.

Early peptide synthesis and even translation may have also occurred over a broad pH range. The peptidyltransfer reaction that takes place at the heart of the ribosome does not involve acid‐base catalysis and so is relatively pH‐insensitive.^145^ A considerable decrease in peptide bond formation is observed only at pH<6.5 due to inactivation of the attacking amino group of the A‐site aa‐tRNA by protonation.^146^, ^147^ Notably, the activation of amino acids by aminoacetylation, a key step in protein biosynthesis, can also be catalyzed by RNA under acidic conditions: Kumar et al. reported the selection of a calcium‐dependent ribozyme capable of activating amino acids in this manner, with an optimal of pH 4.0–4.5.^148^

## 4 Heat tolerance of nucleic acid catalysis

**Figure 6:**
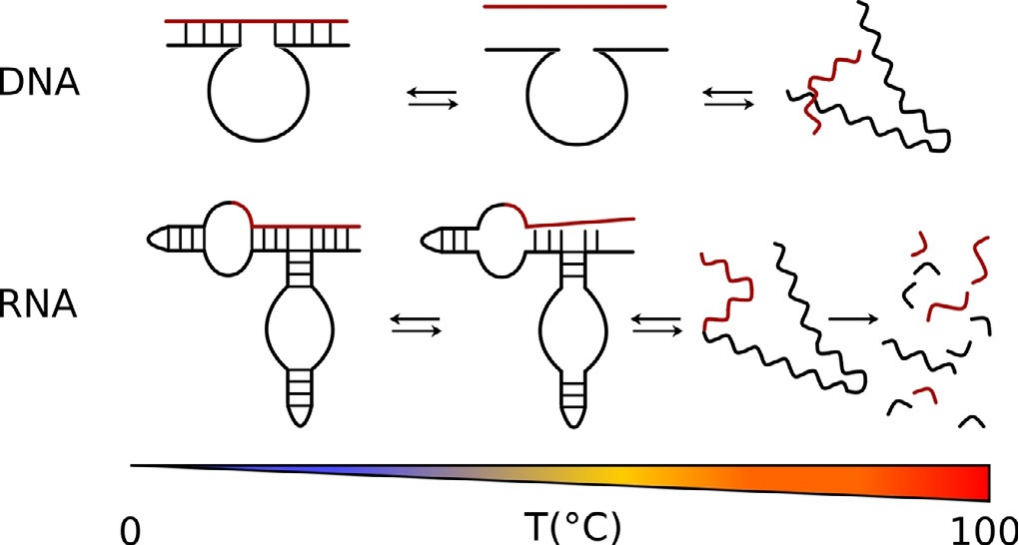
Stability of (deoxy‐)ribozymes with increasing temperature. In aqueous environments, low and moderate temperatures support folding of typical secondary and tertiary DNA and RNA structures. Higher temperatures generally support the reversible melting and the resulting formation of unfolded single‐stranded nucleic acids. However, the individual melting points and pathways are strongly dependent on the overall number and strength of tertiary and secondary interactions, as well as the concentration of counter‐ions. Generally, hybridization of RNA is stronger than that of DNA. High temperatures also increase the rate of spontaneous and irreversible RNA backbone hydrolysis, which is typically not the case for DNA.

Temperature is a further critical parameter in nucleic acid catalysis and stability (Figure 6). As for proteins, reaction rates increase with increasing temperature, until the point at which activity falls due to denaturation. In the absence of magnesium, the duplex melting temperature (*T_m_*) of nucleic acids is sufficiently low to reduce the catalytic potential at even slightly elevated temperatures. In addition, the faster reaction kinetics at elevated temperatures are offset by the increasing rate of phosphodiester hydrolysis, especially in the presence of divalent metal cations such as magnesium as discussed above, which prevents sustained catalysis.

### 4.1 Prebiotic temperatures and thermophilic RNAs

Temperature estimations of the early Earth are a matter of debate. Several lines of evidence exist that support a hot climate during the Archean eon, 4 to 2.5 billion years ago, by which point the Earth's crust is thought to have cooled sufficiently to allow for the dawn of life. Based on oxygen and silicon isotope analyses in sedimentary rocks,^52^, ^54^, ^149^ turbidity current deposits that suggest a possible low viscosity ancient ocean,^55^ and the progressively decreasing thermostabilities of resurrected ancestral proteins,^150^ Archean surface seawater temperatures have been interpreted to range between 60 °C and 80 °C. In contrast, temperatures below 40 °C at the surface have also been proposed based on evidence including deuterium and phosphate isotope analyses,^56^, ^57^ and Archean glacial deposits suggest the presence of ice caps.^58^ Indeed, more recent 3D climate‐carbon models by Charnay et al. predict global mean temperatures between around 8 °C (281 K) and 30 °C (303 K) 3.8 billion years ago, suggesting that cold and even frozen environments may have been present on early Earth.^151^ Hydrothermal vent temperatures are highly variable, with gradients from the hot interior (>350 °C) to much colder seawater (or surrounding surface freshwater).^51^ This precludes the occurrence of biochemical processes on or near to the surface of the vent, particularly given that the function of typical mesophilic nucleic acid enzymes is lost above ≈70 °C, but conditions in the immediate surroundings may have been rather more amenable.

Despite the temperature sensitivity of RNA, living systems have adapted to survive at extreme temperatures. Comparison of homologous ribozymes in mesophilic and thermophilic organisms reveals how sequence adaptations can lead to higher temperature stability. A study on RNase P homologs in mesophilic and thermophilic bacteria by Pan et al. observed that folding was more cooperative for thermophilic RNA, and the folding pathway proceeded via a different set of intermediate structures despite the high similarity of the final states.^152^ Further work revealed that the thermophilic homolog possesses several mutations that increase its stability by increasing GC content and eliminating non‐canonical base pairs.^153^ In addition, insertions in diverse motifs throughout the thermophilic homolog structure increase tertiary interactions and folding cooperativity while creating a more densely packed core.

### 4.2 In vitro selection of thermophilic nucleic acid enzymes

Several reports focusing on heat adaptation of nucleic acid enzymes to higher temperatures have been published. Guo et al. used directed evolution to select for thermally stable variants of the *Tetrahymena* ribozyme.^154^ A family of temperature stable variants were identified, which were slower than the original ribozyme but had 10.5 °C higher melting temperatures. Whilst the consensus sequence of this family contained nine point mutations, only one served to strengthen the helical secondary structure. The remaining 8 mutations increased tertiary interactions between adjacent motifs, thus improving the packing of the ribozyme structure and presumably favoring active conformations.

Saksmerprome et al. discovered highly thermostable variants of the HH ribozyme.^155^ Through in vitro selection, two groups of minimal HH ribozymes were isolated that exhibited trans catalytic activity at elevated temperatures due to strong tertiary interactions between terminal loops and internal bulges that strengthen ribozyme folding and ribozyme‐substrate binding. High thermal stability may also be achieved without dedicated selection experiments: Vazquez‐Tello et al. discovered that the SMα1 HH ribozyme found in the human parasite *Schistosoma mansoni* HH ribozyme is most active at ≈70 °C in vitro without additional sequence optimizations.^156^ Moreover, the same ribozyme can also be successfully cloned and expressed in the thermophile *Thermus thermophilus* where it catalyzes efficient cis‐ and trans‐ cleavage of mRNA in vivo at temperatures up to 80 °C. In this case, temperature modulates the rate limiting steps of the reaction: at 37 °C, catalysis is limited by substrate dissociation, whereas at high temperature RNA degradation, ribozyme‐substrate association, and secondary structure denaturation limit activity.

DNAzymes capable of high temperature catalysis have also been obtained by in vitro selection. Nelson et al. selected a range of Zn^2+^‐dependent RNA‐cleaving DNAzymes with activity at 90 °C.^157^ The selected sequences share little sequence similarity with other metal dependent DNAzymes, and only slightly enhance cleavage above background levels. Interestingly, no secondary structural features are predicted in the selected sequences at 90 °C, implying that the DNAzyme is capable of binding Zn^2+^ and maintaining catalysis with minimal secondary structure.

These studies demonstrate that the catalysis of nucleic acids can be retained at elevated temperatures. Temperature adaptation in ribozymes is generally achieved through additional RNA‐RNA interactions stabilizing both the catalytically active conformation and RNA‐substrate interactions, allowing activity to be sustained up to 80 °C. These adaptive mechanisms may generally also decrease the M^2+^ dependency of nucleic acid folding and catalysis, which, in the case of RNA, helps to reduce degradation. More work investigating the stabilization of more primitive, short ribozyme systems is required to examine the range of temperatures that permit the emergence or even self‐replication of functional RNAs at increased temperatures. DNA is more resistant to degradation than RNA, so selected DNAzymes can operate at up to 90 °C by relying on metal cofactor binding rather than the maintenance of a well‐folded active site. It is as yet unknown whether such systems are limited to simple reactions such as substrate cleavage.

## 5 Pressure as a modulator of nucleic acid catalysis

In addition to temperature and pH, hydrostatic pressure is also a potentially important environmental factor when considering oceanic or subterranean origins of life. High‐pressure conditions are typically defined as 10 MPa or greater, corresponding to a water depth of 1000 m or more. 88 % of the volume of modern oceans may be considered high pressure, with an average pressure of 38 MPa and a maximum on the abyssal plane of 110 MPa.^158^ Thus, any model of abiogenesis that includes deep‐sea vents must account for hydrostatic pressure, which often has profound effects on biological systems by changing the balance of intermolecular interactions. Long‐range interactions such as Van der Waals forces and salt bridges become weaker under compression, and shorter interactions such as hydrogen bonds are favored. Under pressure, systems shift towards low volume states in accordance with Le Chatelier's principle. In proteins, dissociation and unfolding is associated with a large negative volume change (−30 to −110 mL mol^−1^), whilst the DNA double helix dissociation has a positive Δ*V* of 1–5 mL mol^−1^.^159^, ^160^, ^161^ This leads to dissociation and unfolding of protein systems as hydrophobic surfaces become hydrated, but nucleic acid structures that are dependent on hydrogen bonding are stabilized. The double helical forms of DNA and RNA are typically stabilized by pressure, with a concomitant increase in melting temperature and no major structural changes other than slight structural distortion due to compression of hydrogen bonding interactions.^162^, ^163^ The stabilizing effect is dependent on solution ionic strength and *T_m_*, with duplexes that melt below 50 °C being destabilized by pressure and those melt above 50 °C being stabilized.^159^ Certain non‐canonical nucleic acid structures, such as the DNA G quadruplex, exhibit negative Δ*V*s and melt under pressure.^161^

RNA structures are also remarkably stable under high hydrostatic pressure: few structural changes are observed in tRNA^Phe^ up to 1 GPa.^164^ Some RNA structures, such duplexes consisting of A‐U base pairs, are slightly destabilized by pressure, and more critically the formation of tertiary interactions and docked conformations required for ribozyme catalysis may be disfavored due to positive activation volumes.^165^, ^166^ Indeed, the observed rate of cleavage (*k_obs_*) and overall equilibrium constant of HP ribozyme self‐cleavage decreases with increasing pressure.^166^, ^167^ However, despite the overall retardation of the reaction, the actual self‐cleavage step is accelerated by hydrostatic pressure and the decrease in rate is attributed to the positive activation volume of docking between catalytic loops.^168^ The overall yields of RNA strand cleavage by certain hairpin (HH) ribozymes are improved by high hydrostatic pressure, which can even potentiate catalysis in the absence of the Mg^2+^ typically required for cleavage under ambient pressure.^169^, ^170^ Whilst the hammerhead (HH) ribozyme also has a positive activation volume associated with a transition to an active conformation (although significantly smaller than for HP ribozyme), no observable Δ*V* is associated with the cleavage reaction itself.^171^ Molecular dynamics simulations have demonstrated that enhanced hydrogen bonding interactions in the core of the HP and HH ribozymes are responsible for an enhancement in the rate of cleavage under hydrostatic pressure.^172^ The effect of hydrostatic pressure appears to extend to deoxyribozyme catalysis: the 10–23 DNAzyme was shown to be active under pressure in the absence of magnesium, albeit with reduced overall yield.^169^

These studies demonstrate that hydrostatic pressure can promote nucleic folding and compensate for a lack of magnesium in certain nucleic acid catalysts. The increase in melting temperature associated with pressurization could permit increased reaction temperatures for weakly folding systems, and be used to avoid Mg^2+^‐catalyzed degradation of RNA. When considering undersea environments, the resistance of nucleic acid to pressure‐induced denaturation lends support to a nucleic acid‐based origin of life, especially when considering the drastic effect of such conditions on protein folding.

## 6 Activity enhancement by freezing, evaporation and presence of organic solvents

Apart from the typical physicochemical parameters such as pressure, ionic conditions, pH and temperature described above, more exotic environmental conditions can strongly influence nucleic acid catalysis. A notable example is the extraordinary effect of dehydrating conditions on ribozyme and deoxyribozyme catalysis induced by freezing, evaporation, or the presence of organic solvents.

### 6.1 Freezing and dehydration induced ribozyme catalysis

The discovery that freezing or evaporation can enhance or even trigger ribozyme catalysis was serendipitous. The first reports of (undesired) HH ribozyme activity at sub‐zero temperatures came from investigations of the autocatalytic processing of dimeric tobacco ringspot virus satellite RNA (STobRV RNA) by Prody et al.^12^ The authors reported difficulties during long‐term storage of dimeric STobRV RNA due to self‐processing into monomers during one week of storage of the RNA at −20 °C as a precipitate in 67 % ethanol. Similar observations of “unwelcome” RNA cleavage in hairpin ribozyme/yeast‐mRNA constructs during repeated freezing and thawing were later also reported by Donahue and Fedor.^173^ The first systematic investigation of this effect was carried out in 1998 by Kazakov et al., who reported efficient freezing‐induced self‐ligation of the hairpin (HP) ribozyme even in absence of divalent metal ions such as Mg^2+^, which are usually indispensable for catalysis in low‐salt conditions.^174^ Kazakov and his co‐workers later expanded their work, and showed that alcohol‐induced dehydration and simple evaporation also induced M^2+^‐independent RNA ligation by HP ribozymes in both trans and in cis, while disfavoring the reverse cleavage reaction.^175^, ^176^, ^177^ While divalent metal ions were irrelevant for the freezing‐induced ligation, monovalent ions had a strong impact on ligation yields. In particular, sodium salts of acetate‐phosphate‐borate mixtures, EDTA, and acetate/LiCl led to increased ligation yields.

**Figure 7:**
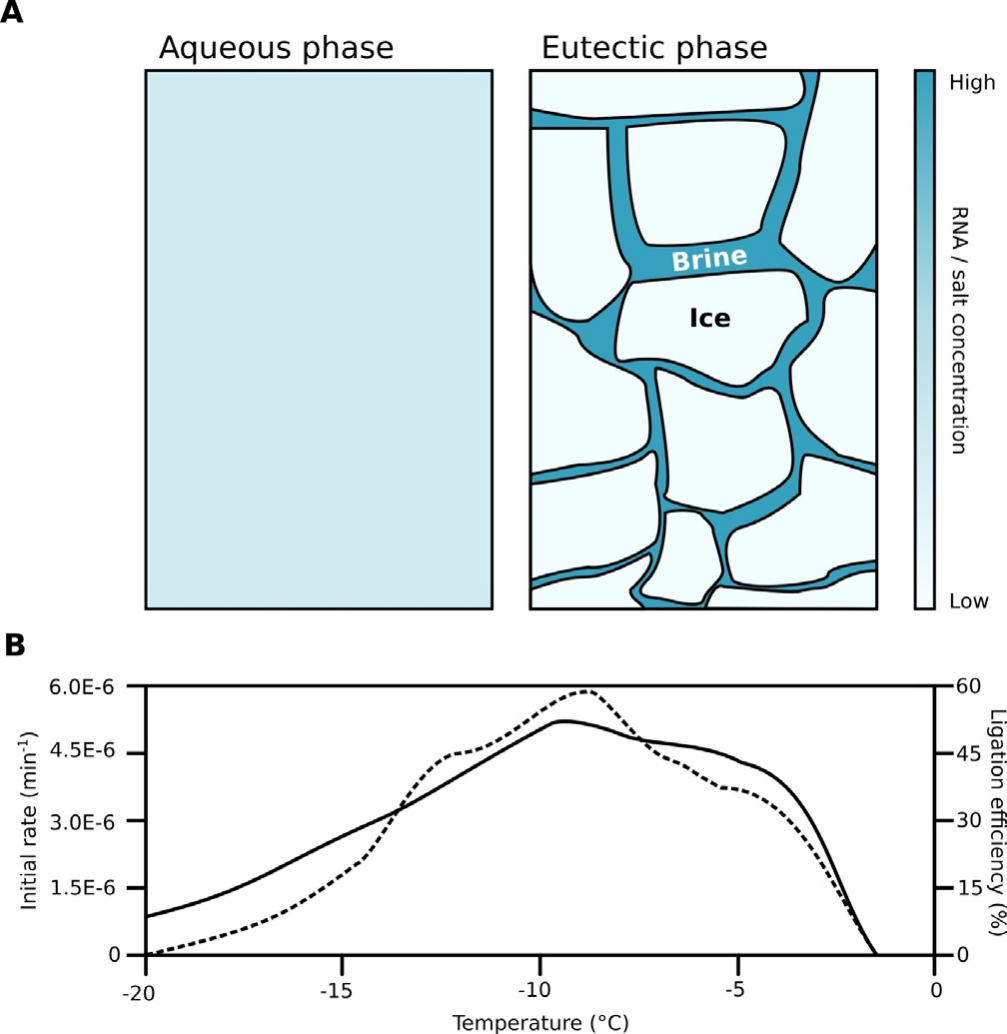
Water ice above the eutectic point is a benign reaction environment for nucleic acids catalysts. A) Schematic showing morphology of eutectic ice phase and relative changes in solute concentration on freezing. The left panel illustrates a dilute aqueous system in an unfrozen state. The right panel shows a partially frozen aqueous solution (e.g. a binary NaCl‐water system containing RNA) above the eutectic point. Solutes in the mother liquor (dark blue) are concentrated as a large fraction of almost pure H_2_O is sequestered in the ice crystals (light blue). This concentration effect leads to a decreased freezing point of the mother liquor and crystal growth stops when the equilibrium between the ice phase and the liquid phase has been reached. B) Illustrated variation in rate (dashed line) and ligation efficiency (solid line) of the HP ribozyme (excess substrate concentration) in a partially frozen, dilute buffer solution (25 mm NaCl, 1 mm Tris⋅HCl pH 7.5).^175^ Both ligation rate and yields are optimal between −4 °C and −12 °C. At lower temperatures, the low thermal energy available in the system makes it difficult to surmount the activation barrier for the reaction. At temperatures approaching 0 °C, melting of the ice inactivates hairpin ribozyme catalysis in absence of Mg^2+^.

A first conjecture as to why monovalent salts are important for HP ribozyme catalysis under frozen conditions is provided by previous studies, which have shown that the absence of M^2+^ can be compensated by high concentrations (>1.5 m) of monovalent cations.^178^ As already discussed above, several of the small nucleolytic ribozymes such as the HP, HH and VS ribozymes are not obligate metalloenzymes (i.e. metal ions are not involved directly in catalysis) but rely on nucleotide‐mediated general acid base catalysis. M^2+^ ions in dilute aqueous solution are still vital for tertiary RNA folding and stabilization of the active conformation.^179^, ^180^ The high concentrations of monovalent cations required to substitute for divalent metal ions are readily available in the aqueous phase of water‐ice mixtures at temperatures above the eutectic point, in which the crystallization of nearly pure water crystals highly concentrates the remaining aqueous phase (Figure 7).^181^

The activation of the HP ribozyme by the high salt concentration in eutectic brine does not at first seem to explain the alcohol‐induced activation of catalysis, since the typical alcohol concentrations used to trigger ribozyme catalysis are not sufficient to co‐concentrate or precipitate monovalent counter‐ions.^176^, ^182^ However, high concentrations of organic molecules such as primary alcohols or polyethers decrease the dielectric constant of the solvent, thereby strengthening cation‐RNA interactions.^183^, ^184^ Thus, M^2+^‐independent ribozyme catalysis in presence of primary alcohols or poly(ethlyene glycol) (PEG) might, as in freezing, be at least partially due to the enhanced RNA‐Na^+^ interactions that can compensate for the missing divalent metal ions.^176^ Indeed, even under normal (aqueous) concentrations, ethanol at concentrations above 30 % significantly increases the Mg^2+^‐dependent activity of ribozymes and mitigates the effects of destabilizing mutations, although higher levels of ethanol in the presence of Mg^2+^ diminishes this activity, presumably due to RNA aggregation.^185^, ^186^, ^187^ In addition to enhancing ion‐ion interactions, dehydration induced by high levels of ethanol or PEG could also support ribozyme activity by promoting the formation of A‐form helices (and therefore the catalytic loop structures of ribozymes defined by adjacent helical segments).^186^, ^188^

Kazakov et al. also reported that HP ribozyme‐catalyzed ligation during evaporation is considerably improved by the presence of PEG, which had no impact on ligation under aqueous or frozen conditions or ethanol‐induced ligation. The authors concluded that PEG might decrease the rate of evaporation, thereby extending the windows of partial dehydration where the water activity is still sufficient to allow hairpin ribozyme catalysis.^189^ The notion that at least some minimal hydration is required for HP ribozyme catalysis is also in agreement with the reports by Seyhan and Burge, who found that low but non‐zero levels of water activity are required for HP and HH ribozyme catalysis in dry RNA films. Intriguingly, hydrated RNA films support cis and trans catalysis over a broad range of temperatures between −70 °C and 37 °C (and probably above), which has potential implications for RNA catalysis under prebiotic conditions.^190^

The formation of active ribozyme conformations in the absence of divalent metal ions can be induced by conditions that promote electrostatic shielding and RNA compaction, such as partial dehydration, up‐concentration of monovalent cations, or reduced dielectric constant. Furthermore, the effective increase in RNA concentration during freezing facilitates RNA‐RNA association, even from very stable monomeric structures,^191^ and has been shown to induce the stretching and alignment of single stranded DNA, which in turn enables its adsorption onto a variety of surfaces.^192^

Freezing favors ligation in reversible transesterification reactions, even from highly fragmented ribozymes.^175^, ^193^, ^194^ Freezing can enable highly thermodynamically disfavored reactions, such as ligation of monomeric 2′, 3′‐cyclic nucleoside monophosphates to a free 5′ end of RNA.^195^ While this reversal of exonucleolytic cleavage has an equilibrium constant of ≈2.2 m ^−1^ under aqueous conditions (at 0 °C^196^), it can be decreased ≈20‐fold by freezing to −9 °C in the presence of 25 mm NaCl and 10 mm MgCl_2_, enabling quantitative non‐canonical 3′‐5′ nucleotidyltransfer of RNA.

Both HP and HH ribozyme ligation yields strongly benefit from repeated freeze‐thaw (FT) cycling.^175^, ^193^, ^194^ This effect can even be used to enable the in trans assembly of long structured RNAs, such as the ≈200 nt RNA polymerase ribozymes, from fragments between 20–30 nt.^193^ The beneficial effects of FT cycles are likely the result of reducing the propensity of small ribozymes to form inactive or poorly active ribozyme‐substrate complexes that attenuate bulk catalysis. Repeated freezing and thawing leads to periodic disruption and re‐formation of both active and unproductive complexes (in the absence or at low levels of M^2+^) thereby providing unproductive complexes a “second chance” at catalysis.

Attwater et al. demonstrated the beneficial effects of a frozen environment on strictly M^2+^‐dependent ribozymes such as the R18 RNA polymerase, which catalyzes templated primer extension using nucleoside triphosphates.^197^, ^198^ The cold environment considerably extends the lifetime of the polymerase, whilst the concentrating power of freezing above the eutectic temperature enables RNA polymerase activity even at extremely low (unfrozen) starting concentrations of RNA, NTPs and Mg^2+^ salts. The authors also investigated the impacts of different negative counter‐ions to Mg^2+^, and found that they markedly influence activity, presumably due to their influence on the eutectic freezing point, which dictates the concentrating effect of the eutectic brine. The ice microstructure has been shown to provide a quasi‐cellular compartmentalization enabling robust phenotype‐genotype linkage, which is one of the key requirements for Darwinian Evolution.^198^ Indeed, this in‐ice compartmentalization was later used by Attwater et al. to isolate a cold‐adapted RNA polymerase ribozyme with considerably increased activity compared to ribozymes selected at ambient temperatures.^199^ Recently, Attwater et al. were also able to evolve an ice‐adapted RNA trinucleotide polymerase ribozyme that is able to copy its own 170 nt catalytic subunit via the ligation of its almost exclusively triplet‐synthesized fragments.^200^

### 6.2 Freezing and dehydration induced deoxyribozyme catalysis

**Figure 8:**
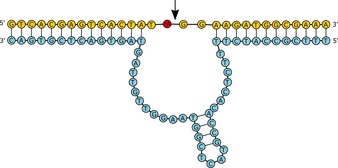
Secondary structure of the EtNa DNAzyme.^201^ The substrate strand is shown in yellow, with the ribonucleotide marked in red. The cleavage site is marked by an arrow.

Zhou et al reported the isolation of the DNAzyme EtNa (Figure 8) from a random DNA library, which is specifically adapted to catalyze RNA cleavage in concentrated organic solvents containing only monovalent Na^+^.^201^ EtNa shows a rate enhancement of up to 1000‐fold in 54 % ethanol compared to water in presence of 4 mm NaCl, and is completely independent from divalent metal ions. The EtNa RNA cleavage rate can be directly used as a biosensor for the precise measurement of alcohol levels in spirits such as whisky or vodka. Interestingly, EtNa activity drastically decreases at ethanol concentrations beyond 72 % (v/v) ethanol, where the B‐form helix of DNA is converted into the A‐form that (in contrast to ribozymes) seems to be incompatible with the formation of the active DNAzyme conformation. Given that EtNa shows cooperative binding of and activation by Ca^2+^ (in contrast to Mg^2+^)^202^ it can also be used as an ultrasensitive biosensor capable of detecting Ca^2+^ levels down to 1.4 μm Ca^2+^.^203^ Eutectic freezing can also activate EtNa, while other DNAzymes that depend on divalent or trivalent metals are inhibited under these conditions.^204^ This again highlights the interchangeability of freezing, organic dehydration or evaporation to achieve activation of metal‐independent nucleic acid catalysts.

### 6.3 The potential of wet‐dry cycles

The remarkable ability of dehydration to potentiate ribozyme function suggests that such conditions may have been important to the emergence of replicating RNA. Wet‐dry cycles, perhaps driven by day‐night cycles or geothermal activity on early Earth, have been proposed as possible drivers of the emergence of function. Viscous environments formed by water evaporation facilitate non‐enzymatic RNA replication cycles slowing reannealing and thereby circumventing strand inhibition.^205^ This effect was used by He et al. to form a HH ribozyme by the enzymatic ligation of short fragments, which was functional following dilution in water.^206^

**Figure 9:**
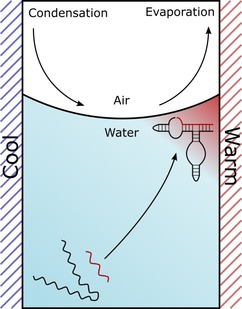
Schematic of a heated rock pore. Thermal gradients at an air–water interface can result in an environment which up‐concentrates a variety of components including ribozymes and ions.^207^ The improved ribozyme catalysis is most likely the result of local high magnesium and RNA concentrations at the interface. However, direct dehydration of the RNA at the temporally dried interface on the warm side (red) may also contribute to activity. Depending on the geometry of the system, evaporated water condenses at the cold side. The forming water droplets can fall back into the mother solution and wash off the dried components. This can lead to microscopic wet‐dry cycles.

Wet‐dry cycles can also be produced by the application of thermal gradients at an air–water interface (Figure 9). The resulting environment up‐concentrates a variety of components including RNA precursors and oligonucleotides, enabling a compelling variety of prebiotically important processes including precursor crystallization and phosphorylation.^207^ Furthermore, the same environment substantially improves ribozyme catalysis and encapsulation within lipid vesicles. The improved ribozyme catalysis is primarily the result of local high magnesium and RNA concentrations at the air–water interface, but dehydration may also be significant.

## 7 Ultraviolet light

Exposure to UV radiation presents a challenge to the survival of prebiotic nucleic acids, and is often raised as a major problem in any RNA world scenario due to the elevated levels of surface UV radiation compared to the present day.^208^, ^209^ Absorption of ultraviolet photons by nucleobase aromatic rings leads to an excited and highly reactive electronic state, which can give rise to chemical lesions such as adenine cycloaddition to A or T in DNA,^210^ as well as the formation of cyclobutane pyrimidine dimers in both DNA and RNA (Figure 9).^211^ The effect of UV damage on nucleic acids has been investigated extensively (reviewed by Wurtmann and Wolin),^211^ and UV‐induced RNA‐RNA crosslinking is now an established method for characterizing tertiary or quaternary RNA structure.^212^

Despite its deleterious effect of nucleic acids, ultraviolet radiation has been observed to promote prebiotic chemical reactions that yield ribonucleotides^213^, ^214^, ^215^ and amino acids,^216^ and has been proposed as a possible energy source to drive prebiotic chemistry on early Earth.^217^ As such, UV radiation could provide an important link between prebiotic chemistry and emergence of an RNA World, but only if radiation levels required to drive such prebiotic reactions can be reconciled with nucleic acid stability under irradiation. Key questions are: To what degree can nucleic acid enzymes sustain photodamage and retain function? Is it possible for nucleic acid enzymes to adapt to strong UV environments?

Despite the well‐documented exploration of UV‐induced nucleic acid damage, relatively few insights are available regarding the role of UV exposure on functional RNA (or other nucleic acid) enzymes. This may be in part due to a complex interplay between UV radiation and other factors influencing RNA catalysis, such as the presence of metal ions. When exposed to UV radiation, tobacco mosaic virus (TMV) RNA accumulates lesions in the form of uridine hydrates and pyrimidine dimers. However, in the presence of magnesium the rate of accumulation was approximately one‐third than that in water, implying that folded RNA is more resistant to UV radiation damage than the unfolded random coil.^218^

The influence of structure and conformation on nucleic acid UV sensitivity was further demonstrated by Kundu et al., who reported an unexpected discrepancy between the UV sensitivities of dTdT dinucleotides in either RNA or DNA hairpins.^219^ dTdT dinucleotides embedded in DNA hairpins, which typically adopt a B‐form double strand, were susceptible to the formation of photolesions, whilst those in A‐form RNA hairpins were protected from damage. The authors also demonstrated that the photosensitivity of the dTdT dinucleotides is modulated by sequence context, with the accumulation of dTdT lesions reduced by neighbouring dA nucleotides, and almost completely inhibited by neighbouring dG nucleotides.^219^ It is fascinating that nucleic acids can gain UV resistance simply by adopting a more compact helical conformation, and the sequence dependence of UV photosensitivity suggests that adaptation of nucleic acids to strong UV environments could be possible. Despite this, it must be noted that the effect of UV exposure on functional RNA in vivo typically decreases function.^220^, ^221^, ^222^

Recently, Saha and Chen monitored the function, folding, and kinetics of RNA aptamers that bind conditionally fluorescent ligands in vitro following UV induced photodamage.^223^ One aptamer, Spinach2, retained significant levels of fluorescence after UV exposure compared to the malachite green aptamer. This may be because a large portion of the Spinach aptamer's binding site is comprised of a photostable G‐quadruplex. Single‐stranded binding regions were found to be more UV sensitive, confirming that duplex formation is protective against UV radiation,^223^, ^224^ and that UV sensitivity significantly depends on folding and conformation.^219^

**Figure 10:**
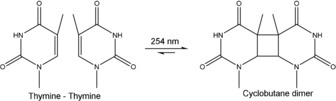
Photodimerization of adjacent thymine nucleotides (dTdT) following exposure to UV light. This photolesion is repaired by the UV1C photolyase DNAzyme, whilst the Sero1C DNAzyme can repair a more diverse range of dimers including thymine, uracil, and several deoxypyrimidine–ribopyrimidine chimeras.^225^, ^227^

While UV irradiation has been generally demonstrated to have a detrimental on functional nucleic acids, some examples of UV‐dependent nucleic acid catalysts have been reported. Chinnapen and Sen reported the in vitro selection of a DNAzyme with photolyase activity, UV1C, from a pool of random sequences.^225^ UV1C is capable of repairing dTdT dimers caused by UV exposure, and requires UV light to function in a manner similar to extant protein photolyase enzymes (Figure 10). The authors later demonstrated that a G‐quadruplex near the substrate binding site functions as both an antenna to absorb UV photons and as an electron source for the repair reaction.^226^ Intriguingly, a serotonin cofactor dependent photolyase DNAzyme was later selected, which is able to repair both thymine and uracil dimers on ribose and deoxyribose backbones.^227^ The discovery that nucleic acids can both harness UV radiation and use this energy to repair photodamage is important, as it provides a mechanism for early replicating systems to survive heavy UV irradiation on Early Earth. In the absence of such a mechanism, early replicators would have to depend on environmental protection from UV radiation, such as the protective effect of montmorillonite clay particles,^228^ or shielding by oceanic UV absorbers.^208^

## 8 Conclusion and perspectives

The activities of both ribozymes and deoxyribozymes are compatible with a broad range of potentially prebiotic conditions. Despite being less versatile and powerful than protein‐based catalysis, nucleic acid catalysts are capable of escaping irreversible aggregation, while also tolerating or even benefiting from much harsher conditions such as freezing, drying or dehydration. Moreover, nucleic acid catalysts often require only modest changes in their sequences to adapt to novel challenging conditions such as harsher pH values or higher temperatures, and can often tolerate or adapt to a broad range of different metal ion cofactors. These combined features make them ideal candidates for early biocatalysis, which presumably emerged and remained functional outside the sheltered and constant milieu of the modern cell.

Despite the large body of research, further explorations of nucleic acid enzymes under prebiotic conditions may yield yet more unforeseen properties relevant for abiogenesis, and warrant further investigation. For example, selection experiments under prebiotically plausible conditions beyond aqueous solutions in a modern oxygen‐rich atmosphere could reveal further unexpected catalytic properties of ribozymes. In addition to the factors discussed in this review, other environmental factors such as mineral surfaces,^228^, ^229^, ^230^ pH gradients,^231^ high viscosities^206^ or combination of various different environments may further enhance the functional repertoire of early nucleic acids. For example, the clay montmorillonite inhibits HP ribozyme catalysis, but surface adsorption to this mineral offers protection against UV degradation.^228^ Clay can also enhance recombination ribozymes and favor ligation by preferentially adsorbing longer RNA strands.^230^ Furthermore, it is possible that heterogeneous complexes such as RNA/peptide complexes or mixed RNA/DNA (or alternative preRNA/preDNA) systems were important forerunners to modern biochemistry, and allowed the catalysis of biochemical or replicative processes that “pure” RNA or DNA systems are presently incapable of.^5^

Finally, it remains essential to further expand far‐from‐equilibrium scenarios to explore different stages of molecular evolution (including nucleic acid catalysis) experimentally under heterogeneous conditions, such as the continuous provision of chemical fuel and/or pH, temperature, or salinity cycles.

## Conflict of interest

The authors declare no conflict of interest.

## Biographical Information

***Hannes Mutschler** received his M.Sc. in biophysics from the Humboldt University of Berlin. His Ph.D. topic was on bacterial toxin‐antitoxin systems at the Max Planck Institute of Medical Research/Heidelberg University, Germany. For his postdoc on origins of life research, he joined Phil Holliger's lab at the Protein and Nucleic Acid Chemistry Division lab at the MRC Laboratory of Molecular Biology in Cambridge, UK. In 2016, he became a MaxSynBio independent research group leader at Max Planck Institute of Biochemistry in Martinsried, Germany. His current research interests include bottom‐up synthetic biology, cell‐free protein synthesis as well as RNA catalysis under prebiotic conditions*.

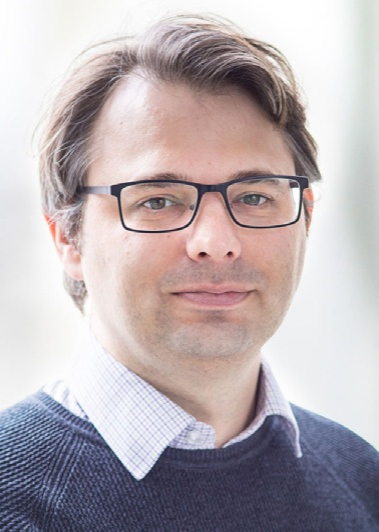

## Biographical Information

***Kristian Le Vay** received his M.Sc. in chemistry from the University of Bristol, UK, and completed his Ph.D. at the Bristol Centre for Functional Nanomaterials. During this time, he designed and developed artificial enzyme systems based on photoactive protein‐nanoparticle conjugates. He is currently a postdoctoral researcher at the Max Planck Institute for Biochemistry. Here, his research encompasses both synthetic biology and origin of life studies, focusing on prebiotic RNA catalysis in unusual environments and the development of dynamic model protocell systems*.

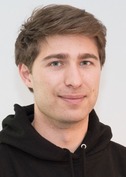

## Biographical Information

***Elia Salibi** completed his B.Sc. in Biology at the University of Beirut, Lebanon. He then received his M.Sc. in Molecular Biology and Genetics from the University of Pavia, Italy. His thesis focused on developing and characterizing a synthetic modular CRISPR/dCas9‐based gene silencing toolbox for Escherichia coli. Currently he is a Ph.D. student at the Max Planck Institute of Biochemistry investigating the role of RNA protocells in the context of the emergence of life*.

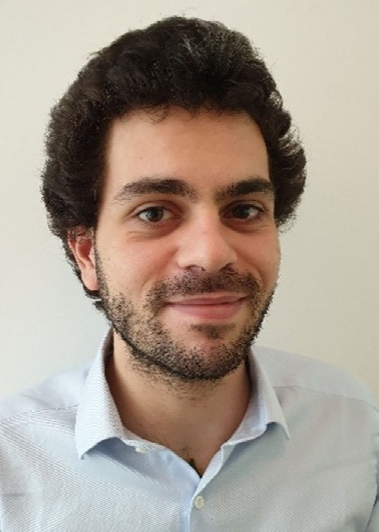

## Biographical Information

***Emilie Y. Song** received her B.Sc. in Biochemistry and Chemistry from the University of British Columbia in Vancouver, Canada. She then completed her M.Sc. in Molecular Genetics at the University of Toronto screening for novel Streptomyces bacterial natural products with potential bioactivity against parasitic worms. She is currently a PhD candidate in the CRC235: Emergence of Life graduate program investigating RNA stability and catalysis in plausible prebiotic environments*.

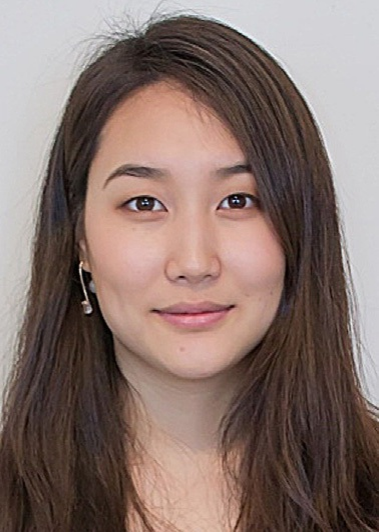

## Supporting information

As a service to our authors and readers, this journal provides supporting information supplied by the authors. Such materials are peer reviewed and may be re‐organized for online delivery, but are not copy‐edited or typeset. Technical support issues arising from supporting information (other than missing files) should be addressed to the authors.

Supplementary
