# Supplementary material for: Nucleic Acid Catalysis under Potential Prebiotic Conditions
Source: Chem Asian J. 2019 Dec 9;15(2):214–30. doi: 10.1002/asia.201901205 (PMC7003795; doi:10.1002/asia.201901205)
Supplement: Supplementary file 1 — Supplementary [file ASIA-15-214-s001.pdf]

Author Contributions

K.L. Conceptualization: Equal; Visualization: Lead; Writing - Original Draft: Lead; Writing - Review & Editing: Lead
E.S. Writing - Original Draft: Equal; Writing - Review & Editing: Supporting
E.S. Writing - Original Draft: Equal; Writing - Review & Editing: Supporting
H.M. Conceptualization: Lead; Funding acquisition: Lead; Project administration: Lead; Resources: Lead; Supervision: Lead; Visualization: Supporting; Writing - Original Draft: Lead; Writing - Review & Editing: Lead.
